# Combination of lymphovascular invasion and the AJCC TNM staging system improves prediction of prognosis in N0 stage gastric cancer: results from a high-volume institution

**DOI:** 10.1186/s12885-019-5416-8

**Published:** 2019-03-11

**Authors:** Jun Lu, Yun Dai, Jian-Wei Xie, Jia-Bin Wang, Jian-Xian Lin, Qi-Yue Chen, Long-Long Cao, Mi Lin, Ru-Hong Tu, Chao-Hui Zheng, Ping Li, Chang-Ming Huang

**Affiliations:** 1 Department of Gastric Surgery, Fujian Medical University Union Hospital, Fuzhou, Fujian Province China; 2 Department of General Surgery, Fujian Medical University Union Hospital, Fuzhou, Fujian Province China; 3 Key Laboratory of Ministry of Education of Gastrointestinal Cancer, Fujian Medical University, Fuzhou, Fujian Province China; 4 Fujian Key Laboratory of Tumor Microbiology, Fujian Medical University, Fuzhou, Fujian Province China

**Keywords:** Gastric cancer, Lymphovascular invasion, Survival, Chemotherapy benefit

## Abstract

**Background:**

This study sought to explore whether lymphovascular invasion can affect the prognosis of patients with stage N0 gastric cancer and to evaluate the survival benefit of adjuvant chemotherapy for such patients.

**Method:**

From January 2006 to December 2011, a total of 2102 gastric cancer patients undergoing radical gastric resection were enrolled in this study. Homogeneity, discriminatory ability, and monotonicity of gradients in the combination of lymphovascular invasion and the 8th edition of the AJCC staging system and the 8th edition of the AJCC staging system alone were compared using linear trend χ2, likelihood ratio χ2 statistics, and Akaike information criterion (AIC) calculations. The Kaplan-Meier method and the log-rank test were used to analyze between-group differences in survival rate.

**Result:**

The median follow-up time of the whole group was 58 months, and the average age of the whole group was 63.9 years (range 21–89 years). The 3-year and 5-year overall survival rates in N0 patients with lymphovascular invasion were lower than those in N0 patients without lymphovascular invasion (3-year OS: 78.3% vs 92.5%, 5-year OS: 70.0% vs 88.3%, *p* < 0.001). A multivariate analysis showed that age (p < 0.001), lymphovascular invasion (p < 0.001), and pT (p < 0.001) were independent risk factors for the prognosis of N0 patients. Compared with the 8th edition of the AJCC staging system alone, the 8th AJCC staging system combined with lymphovascular invasion demonstrated a better linear trend χ2, likelihood ratio χ2 statistics, and AIC value (68.99 vs 58.58, 70.18 vs 58.36, 1473.38 vs 1485.04). In pT3N0M0 patients with lymphovascular invasion, the 3-year and 5-year overall survival rates of the adjuvant chemotherapy group were higher than those of the surgery alone group (3-year OS: 83.3% vs 68.2%, 5-year OS: 72.3% vs 50.0%, *p* = 0.048).

**Conclusion:**

Lymphovascular invasion is an independent prognostic factor in N0 patients. The 8th AJCC staging system combined with lymphovascular invasion can improve the accuracy of the AJCC staging system for N0 patients. Moreover, adjuvant chemotherapy improves the survival of pT3N0M0 patients with lymphovascular invasion.

**Electronic supplementary material:**

The online version of this article (10.1186/s12885-019-5416-8) contains supplementary material, which is available to authorized users.

## Background

Gastric cancer is the fourth leading cause of cancer-related deaths worldwide [1]. Lymph node metastasis is one of the important risk factors for poor prognosis in patients with gastric cancer [2, 3], but the prognosis of patients with lymph node-negative (N0) gastric cancer is not satisfactory [4–6]. Therefore, accurate postoperative staging and prognostic evaluation are particularly important for follow-up and treatment. Current studies have shown that age, sex and tumor size are risk factors for prognosis in patients with N0 disease [7, 8]. However, no consensus has been reached on whether lymphovascular invasion affects the long-term survival of patients with N0 disease. Many researches have confirmed the importance and practicability of the AJCC TNM staging system for the evaluation of the prognosis of gastric cancer patients. Other studies have also improved the accuracy of prediction by improving the AJCC TNM staging system such as the establishment of modified AJCC TNM staging systems combined with age, Lauren classification, lymph node metastasis rate and inflammatory factors [9, 10]. Compared with the AJCC TNM staging system, the AJCC TNM staging system combined with some independent risk factors synthesizes more prognostic information and can comprehensively evaluate patient prognosis [11]. The modified AJCC TNM staging system is more specific for the prognosis of gastric cancer patients in a special population such as patients with stage N0 gastric cancer.

The AJCC TNM staging system plays a guiding role in the selection of adjuvant chemotherapy by clinicians [12]. According to the results from the ACTSGC clinical trial, adjuvant chemotherapy for patients with stage II/III gastric cancer can improve overall survival (HR 0.67, 95% CI 0.540–0.828, [13]. However, differences were observed between the NCCN guidelines and the Japanese gastric cancer treatment protocol in terms of adjuvant chemotherapy for gastric cancer patients with pT3N0M0 disease [12]. The NCCN guidelines recommend that adjuvant chemotherapy be given to patients with stage II/III gastric cancer to improve survival, but patients with pT3N0M0 (stage II) disease were not included based on the indication for adjuvant chemotherapy in the Japanese gastric cancer treatment protocol. Imamura et al. [6] reported the poor prognosis of patients with pT3N0M0 disease and the need for adjuvant chemotherapy to improve overall survival. No consensus has been reached as to whether adjuvant chemotherapy should be administered to patients with pT3N0M0 gastric cancer. The purpose of this study was to explore the relationship among lymphovascular invasion, prognosis and chemotherapy benefit in patients with stage N0 gastric cancer.

## Methods

### Patients

Continuous clinical-pathological data from 3128 patients with gastric cancer who underwent surgical resection from January 2006 to December 2011 at Fujian Medical University Union Hospital (FMUUH) were retrospectively analyzed. The inclusion criteria were as follows: 1) gastric adenocarcinoma confirmed by histopathology, 2) gastrectomy with D2 or D1+ lymphadenectomy,3) complete clinicopathological and follow-up data. Patients with incomplete clinical data (*n* = 195), stage IV (*n* = 323) disease, those who underwent nonradical surgery (*n* = 327) and patients who were lost to follow-up (*n* = 113) were excluded. Patients who underwent neoadjuvant chemotherapy were also excluded from the study (*n* = 68). Thus, 2102 patients were included in the final analysis. Preoperative clinical staging was based on gastroscopy and abdominal computed tomography (CT). Postoperative pathological diagnosis and staging were performed in accordance with the 8th edition of the AJCC TNM staging system. Hematoxylin & eosin (HE) staining was performed to evaluate the presence of venous invasion, and IHC stain for D2–40 was performed using a mouse monoclonal antibody against human lymphatic endothelium antigen to evaluate lymphatic invasion [14]. Patients with N0 stage who were treated with surgery alone were categorized into the surgery alone group (SA-group), and patients with N0 stage who were treated with adjuvant chemotherapy after surgery were categorized into the adjuvant chemotherapy group (AC-group). The study protocol was approved by the ethical committee of the FMUUH.

### Adjuvant chemotherapy

It was suggested that patients with pathological stage II/III be hospitalized for chemotherapy within 4–6 weeks after surgery [12]. Those patients were recommended to receive fluorouracil-based monotherapy or combination chemotherapy with a regimen involving the equivalent of at least one course of treatment after surgery. Most patients with stage II or III gastric cancer in our center usually received fluoride-based adjuvant chemotherapy, including FOLFOX, XELOX, SOX regimen. Ultimately, the decision as to whether a patient receives chemotherapy depends on the patient’s willingness and economic income.

### Follow-up

The endpoint of this study was December 2011 or the time of patient death. Patients were seen at either our outpatient clinic or a local hospital. To determine OS, we calculated the time from the date of surgery to death or the censor date (December 2011). Patients were seen regularly at 3-month intervals during the first 2 year, every 6 months from years 3 to 5, and on a yearly basis thereafter. Generally, physical examination and laboratory tests (such as tumour markers) were performed in each follow-up visit. Examinations including abdominal CT, and endoscopy were performed every 6 months during the first 3 years and annually thereafter. When relapse was suspected, further evaluation, such as PET, whole-body bone scanning and magnetic resonance imaging (MRI), was performed. Recurrence patterns were classified as local recurrence (anastomotic or gastric remnant),lymph node and distant metastasis (peritoneal,hepatic, pulmonary, or other sites of metastatic disease) [15].

### Statistics

Significance was tested using Student’s t-test for continuous variables and χ2 tests for categorical variables. Univariate and multivariate analyses of risk factors for OS after surgery were performed using Cox proportional hazards models, and the results were expressed in terms of HR values and corresponding 95% confidence intervals. OS rates were calculated using the Kaplan-Meier method, and the log-rank test was used to analyze between-group differences in survival rate. The significance threshold was 5%, and all statistical tests were 2-sided. Analyses were performed using Statistical Package for the Social Sciences (SPSS) 23.0 for Windows (SPSS, Chicago, IL, USA).

## Results

### Clinico-pathological characteristics

The clinico-pathological data of the 2102 included patients are shown in Table 1. Out of all patients, 1593 were male (75.8%) with an average age of 63.9 years (range 21–89 years) and 509 were female (24.2%)with an average age of 63.2 years (range 20–91 years). The median follow-up time was 58 months (range 2–111 months). The average tumor size was 4.9 cm, and the average BMI was 21.5 kg/m^2^. Of the 736 N0 patients, 106 had lymphovascular invasion, which accounted for 14.4% of all N0 patients.

**Table 1:** Patient clinicopathological characteristics

Variable	Overall patients (*n* = 2102)	N0 patients (*n* = 736)
Sex		
Male	1593	564
Female	509	172
Age, mean (SD)	63.8, (11.2)	63.5, (11.4)
BMI, mean (SD)	21.5, (3.3)	21.3, (3.1)
Operation Time, mean (SD)	201, (64)	196, (67)
Tumor Size, mean (SD)	4.9, (1.9)	4.6, (2.1)
Tumor Location		
upper third	697	218
middle third	467	126
lower third	938	392
Tumor differentiation		
well differentiated	682	211
moderately differentiated	720	317
poorly differentiated or undifferentiated	700	208
Lymphovascular invasion		
Yes	498	96
No	1604	640
T-stage		
T1	459	356
T2	240	123
T3	504	117
T4	899	109
N-stage		
N0	736	**/**
N1	301	**/**
N2	354	**/**
N3	711	**/**
AJCC stage		
Ia	378	387
Ib	171	123
IIa	187	117
IIb	253	109
IIIa	228	**/**
IIIb	344	**/**
IIIc	532	**/**

### Occurrence of and risk factors for lymphovascular invasion in N0 patients

Additional file 1: Table S1 shows the incidence of lymphovascular invasion according to pT stage. The incidence of lymphovascular invasion in pT1, pT2, pT3, and pT4 disease was 8.3, 13.0, 29.1, and 22.0%, respectively. Univariate and multivariate analyses of lymphovascular invasion in N0 patients showed that only pT stage (HR 3.13, 95% CI (1.75–5.59), *p* < 0.001) was an independent risk factor for lymphovascular invasion (Additional file 2: Table S2).

### Overall survival, recurrence-free survival and disease-specific survival for N0 patients with or without lymphovascular invasion

**Fig. 1:**
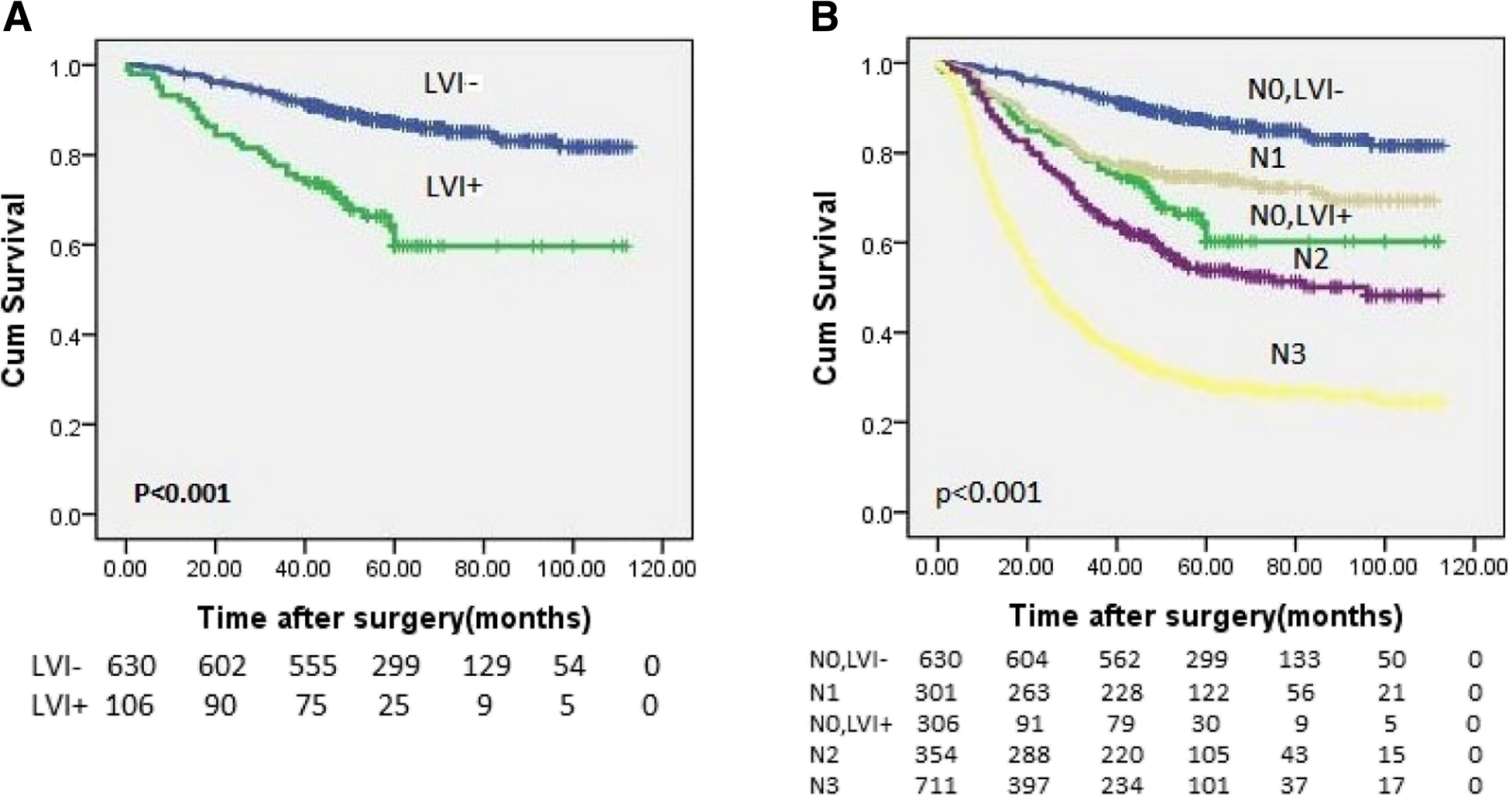
OS curves for gastric cancer patients who underwent radical surgical resection. **a** Log-rank test for overall survival: N0 with LVI- versus N0 with LVI+, *p* < 0.001. **b** Log-rank test for overall survival: N0 with LVI+ versus N0 with LVI-, N1, N2, N3, p < 0.001. *OS: overall survival, LVI: lymphovascular invasion, LVI−/+: negative/positive LVI*

As shown in Fig. 1a, the 3-year and 5-year overall survival rates (3-year OS and 5-year OS) of N0 patients with lymphovascular invasion were significantly lower than those of N0 patients without lymphovascular invasion (3-year OS: 78.3% vs 92.5%, 5-year OS: 70.0% vs 88.3%, *p* < 0.001). Additional file 3: Figure S2. shows the Recurrence-free survival (RFS) and Disease-specific survival (DSS) for N0 patients with or without lymphovascular invasion.(3-year RFS: 76.1% vs 92.0%, 5-year RFS: 64.2% vs 84.8%, *p* < 0.001;3-year DSS: 78.1% vs 93.1%, 5-year DSS: 70.2% vs 87.8%, p < 0.001). The recurrence rate of N0 patients with lymphovascular invasion was 18.5% while 7.8% of N0 patients without lymphovascular invasion occurred recurrence. Among all patients with recurrence, the incidence of distant metastasis was 14.3%, and that of local recurrence was 12.3%.

### Univariate and multivariate analyses for the prognosis of N0 patients

According to the univariate analyses, age, tumor location, lymphovascular invasion, and pT stage were significantly associated with OS (all *p* < 0.05) (Table 2). Moreover, age (HR 2.16, 95% CI (1.39–3.38), p < 0.001), lymphovascular invasion (HR 2.474, 95% CI (1.64–3.72), p < 0.001), and pT stage (HR 3.18, 95% CI (1.82–5.59), p < 0.001) were independently associated with OS in a multivariate analysis (Table 2).

**Table 2:** Univariate and Multivariate analyses of the 5-year OS in N0 patients

Variable		Univariate			Multivariate	
	HR	95% CI	*p* Value	HR	95% CI	p Value
Age			p < 0.001			p < 0.001
age < 55	Ref	/		Ref	/	
55 ≤ age < 65	1.06	0.62–1.79		1.04	0.62–1.80	
age ≥ 65	2.48	1.59–3.86		2.16	1.39–3.38	
Sex			*p* = 0.06			
female	Ref	/				
male	0.63	0.39–1.02				
Tumor location			p < 0.001			
upper third	Ref	/				
middle third	2.47	1.66–3.67				
lower third	1.58	0.94–2.66				
Tumor differentiation			*p* = 0.43			
well differentiated	Ref	/				
moderately differentiated	0.84	0.54–1.29				
poorly differentiated or undifferentiated	0.92	0.58–1.45				
Lymphovascular Invasion			p < 0.001			p < 0.001
no	Ref	/		Ref	/	
yes	3.26	2.21–4.83		2.47	1.64–3.72	
T-stage			p < 0.001			p < 0.001
T1	Ref	/		Ref	/	
T2	1.73	0.93–3.22		1.54	0.83–2.88	
T3	3.6	2.07–6.26		2.62	1.48–4.64	
T4	3.8	2.23–6.73		3.18	1.82–5.59	

### Improvement of the 8th edition of the AJCC TNM staging system

**Fig. 2:**
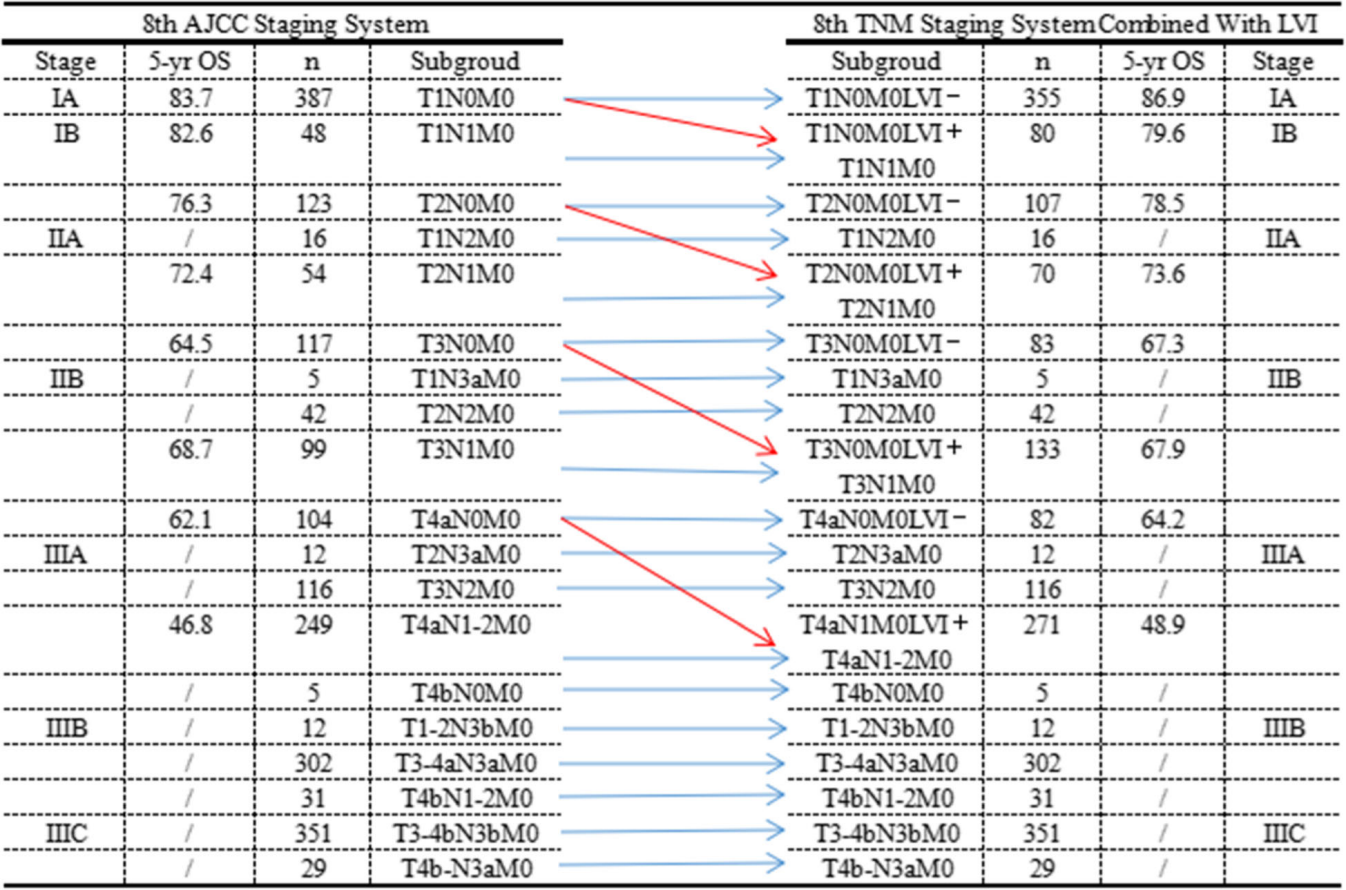
AJCC stage and TNM subgroup distributions of patients according to the 8th edition of the TNM Staging System alone and the 8th edition of the TNM Staging System combined with LVI classification. *LVI: lymphovascular invasion, LVI−/+: negative/positive LVI*

The overall survival rate of N0 patients with lymphovascular invasion was similar to that of N1 patients (3-year OS: 78.3% vs 78.7%, 5-year OS: 70.0% vs 75.1%) (Fig. 1b). The difference between the 8th edition of the AJCC TNM staging system combined with lymphovascular invasion and the 8th edition of the AJCC TNM staging system alone is shown in the Additional file 4: Figure S1. N0 patients with lymphovascular invasion were upgraded to stage N1, while N0 patients without lymphovascular invasion remained in stage N0. Figure 2 shows the changes in the 8th edition of the AJCC TNM staging system combined with lymphovascular invasion compared with the 8th edition of the AJCC TNM staging system alone. In all, 32 patients with pT1N0M0 disease and lymphovascular invasion were upgraded to pT1N1M0, 16 patients with pT2N0M0 disease and lymphovascular invasion were upgraded to pT2N1M0, 34 patients with pT3N0M0 disease and lymphovascular invasion were upgraded to pT3N1M0, and 22 patients with pT4N0M0 disease and lymphovascular invasion were upgraded to pT4N1M0.

### Comparison of the 8th edition of the AJCC TNM staging system combined with lymphovascular invasion and the 8th edition of the AJCC TNM staging system alone

**Fig. 3:**
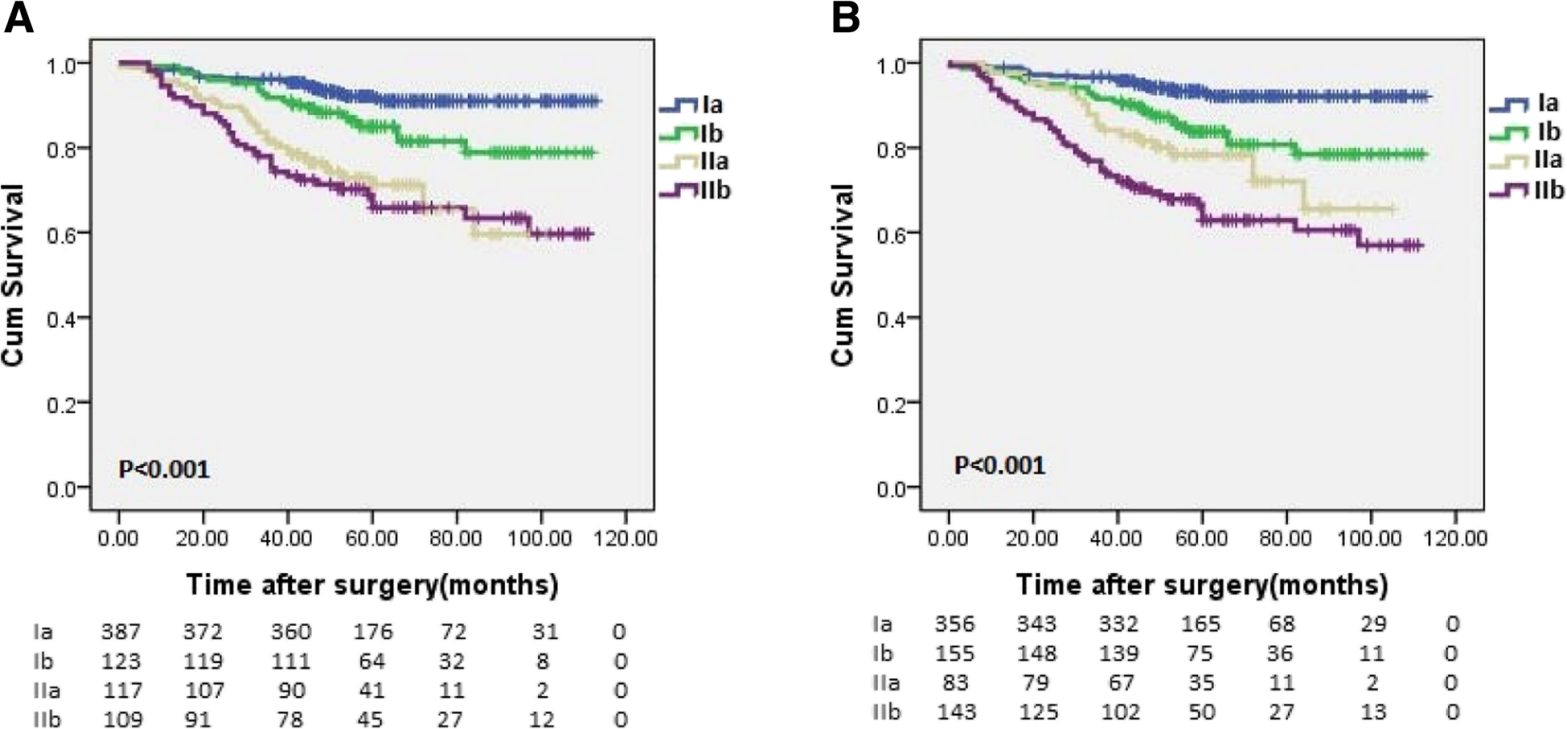
Comparison of survival curves between the 8th edition of the TNM Staging System and alone and the 8th edition of the TNM Staging System combined with LVI. **a** Log-rank test for the 8th edition of the TNM Staging System alone, **b** Log-rank test for the 8th edition of the TNM Staging System combined with LVI, *p* < 0.001

Figure 3 shows the survival curve of the 8th edition of the AJCC TNM staging system combined with lymphovascular invasion and the 8th edition of the AJCC TNM staging system alone. The 8th edition of the AJCC staging system combined with lymphovascular invasion demonstrated a better linear trend χ2, likelihood ratio χ2 statistics, and AIC value (68.99 vs 58.58, 70.18 vs 58.36, 1473.38 vs 1485.04) compared with the 8th edition of the AJCC staging system (Table 3).

**Table 3:** Comparison of the performance of the 8th edition of the TNM Staging System alone and the 8th edition of the TNM Staging System combined with LVI

Classification	Linear trend x^2^	Likelihood ratio x^2^	AIC
8th TNM	58.58	58.36	1485.04
8th TNM + LVI	68.99	70.18	1473.38

### Relationship between lymphovascular invasion and chemotherapeutic benefits in patients with pT1-3N0M0 gastric cancer

**Fig. 4:**
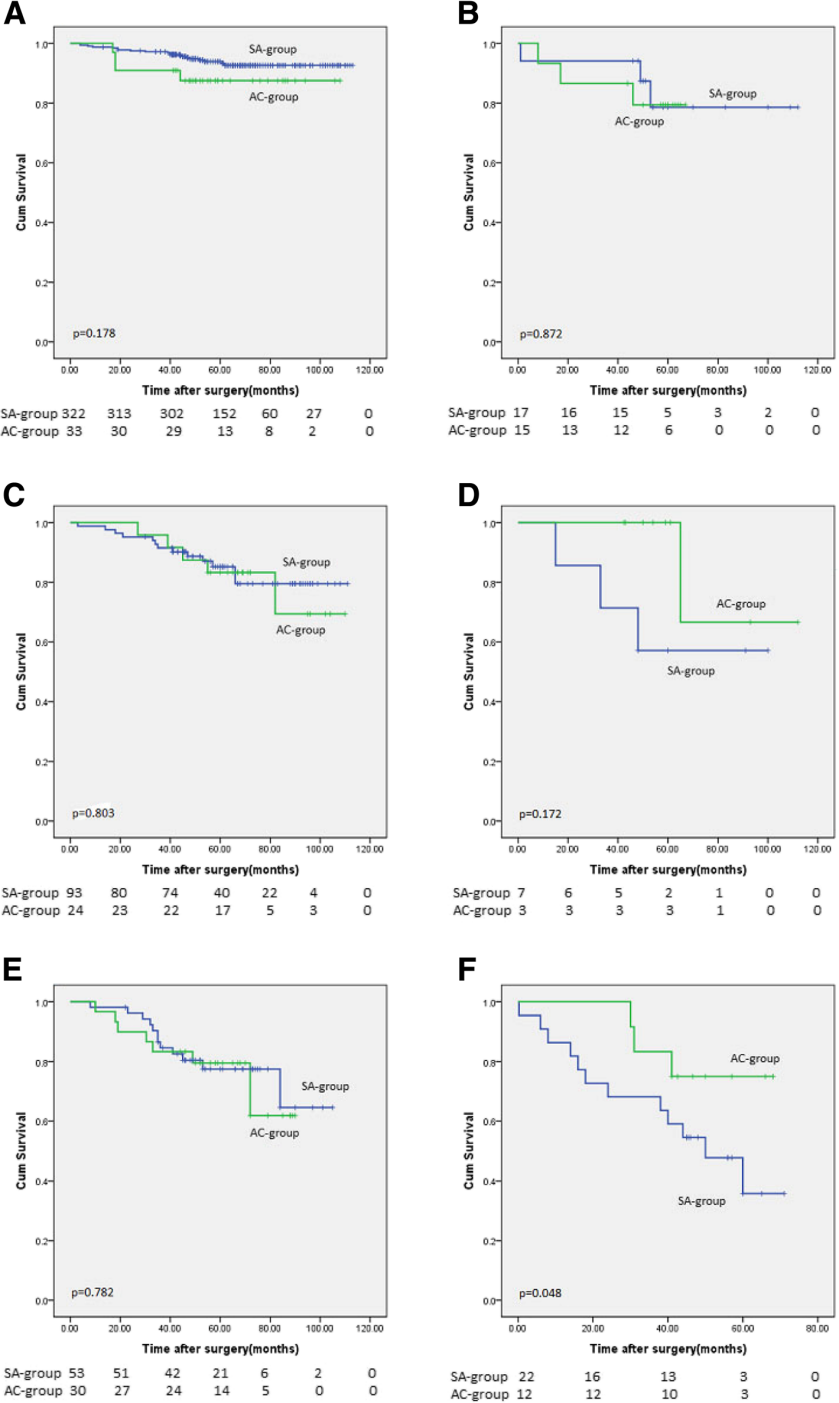
Kaplan-Meier method compares the survival benefit between the SA-group and the AC-group in patients with pT1-3N0M0. **a** Log-rank test for pT1N0M0, LVI- patients: SA-group versus AC-group, *p* = 0.178; **b** Log-rank test for pT1N0M0, LVI+ patients: SA-group versus AC-group, *p* = 0.872. **c** Log-rank test for pT2N0M0, LVI- patients: SA-group versus AC-group, *p* = 0.803; **d** Log-rank test for pT2N0M0, LVI+ patients: SA-group versus AC-group, *p* = 0.172. **e** Log-rank test for pT3N0M0, LVI- patients: SA-group versus AC-group, *p* = 0.782; **f** Log-rank test for pT3N0M0, LVI+ patients: SA-group versus AC-group, *p* = 0.048. *SA-group: surgery alone group, AC-group: adjuvant chemotherapy group. LVI: lymphovascular invasion, LVI−/+: negative/positive LVI*

As shown in Fig. 4, no significant difference was observed in the 3-year OS or the 5-year OS between the adjuvant chemotherapy group and the surgery alone group in pT1-2N0M0 patients without lymphovascular invasion (pT1N0M0, *p* = 0.178; pT2N0M0, *p* = 0.803) and in pT1-2N0M0 patients with lymphovascular invasion (pT1N0M0, *p* = 0.872; pT2N0M0, *p* = 0.172). Compared with the surgery alone group, pT3N0M0 patients without lymphovascular invasion who received adjuvant chemotherapy exhibited a similar survival (3-year OS: 83.3% vs 84.9%, 5-year OS: 80.0% vs 79.2%, *p* = 0.78). In pT3N0M0 patients with lymphovascular invasion, the 3-year and 5-year overall survival rates of the adjuvant chemotherapy group were higher than those of the surgery alone group (3-year OS: 83.3% vs 68.2%, 5-year OS: 72.3% vs 50.0%, *p* = 0.048).

## Discussion

The current AJCC/UICC guidelines do not include lymphovascular invasion as a prognostic indicator of gastric cancer in the TNM staging system. However, studies have shown that lymphovascular invasion is an independent risk factor for survival in patients with gastric cancer [16, 17]. Patients with lymphovascular invasion have been shown to have a worse prognosis. According to the results of Brücher et al., lymphovascular invasion is a strong prognostic factor in patients with esophageal cancer [18]. In view of the prognostic efficacy of lymphovascular invasion, Rahden et al. suggested that lymphovascular invasion should be incorporated into the TNM staging system for gastroesophageal carcinoma in order to improve predictive efficacy [19]. Few studies have been published on lymphovascular invasion in N0 patients. Therefore, we aimed to investigate whether lymphovascular invasion affects the prognosis of N0 patients and the selection of appropriate patients for postoperative adjuvant chemotherapy according to the status of lymphovascular invasion.

In this study, 106 patients had lymphovascular invasion, which accounted for 14.4% of all N0 patients; this was similar to the incidence of lymphovascular invasion reported in related studies [20]. The prognosis of N0 patients with lymphovascular invasion was significantly worse than that of patients without lymphovascular invasion (3-year OS: 78.3% vs 92.5%, 5-year OS: 70.0% vs 88.3%, *P* < 0.001). This may be related to the failure of tumor cells to be completely cleared as well as to the metastasis and recurrence that can occur throughout the lymphatic vessel network. The multivariate analysis indicates that lymphovascular invasion is an independent prognostic factor in N0 patients, which is consistent with the results of many current studies that have suggested that lymphovascular invasion should be included as an indicator in TNM staging.

Many studies have shown that the TNM staging system has important guiding significance for prognostic evaluation and the subsequent treatment of gastric cancer patients. Our results showed that the survival rate of N0 patients with lymphovascular invasion was similar to that of N1 patients. Therefore, N0 patients with lymphovascular invasion were upgraded to stage N1, while N0 patients without lymphovascular invasion remained in stage N0. Moreover, we included lymphovascular invasion as a prognostic indicator in the 8th edition of the TNM staging system. Many studies on the prognostic accuracy of staging systems have agreed that staging systems with lower AIC, higher linear trend χ2, and higher likelihood ratio χ2 statistics have better prognostic accuracy [21, 22]. In the present study, the 8th edition of the TNM staging system combined with lymphovascular invasion was verified by linear trend χ2, likelihood ratio χ2 and AIC value, which improved the ability for prognostic prediction and helped to more accurately evaluate the prognosis of N0 patients.

Currently, the NCCN guidelines suggest that patients with stage II/III gastric cancer receive adjuvant chemotherapy after surgery, but patients with pT3N0M0 (stage II) disease were not included based on the indication for adjuvant chemotherapy in the Japanese gastric cancer treatment protocol. A consensus has still not been reached as to whether pT3N0M0 gastric cancer patients should be treated with postoperative adjuvant chemotherapy. In this study, we found that lymphovascular invasion was closely related to the survival of patients with pT3N0M0 gastric cancer. The prognosis of patients with N0 disease and lymphovascular invasion was significantly worse than that of patients without lymphovascular invasion. In patients with pT3N0M0 disease and lymphovascular invasion, the 3-year and 5-year overall survival rates of the adjuvant chemotherapy group were higher than those of the surgery alone group (*p* = 0.048). Based on this result, we suggest that postoperative adjuvant chemotherapy may improve the survival of patients with pT3N0M0 disease and lymphovascular invasion.

The limitations of this study are discussed below. First, this is a single-center retrospective analysis that requires further prospective studies to confirm the findings. Second, the effects of chemotherapeutics and the number of chemotherapy cycles on prognosis were not analyzed in this study. Finally, the recurrence-free survival of N0 patients was not analyzed in this study.

## Conclusion

Lymphovascular invasion is an independent risk factor for the prognosis of patients with N0 gastric cancer, and the inclusion of lymphovascular invasion is helpful for improving the accuracy of the 8th edition of the AJCC TNM staging system, which is used to determine the stage and prognosis of N0 patients. Finally, adjuvant chemotherapy in pT3N0M0 patients with lymphovascular invasion can improve survival.

## Additional files

Additional file 1: **Table S1**. Lymphovascular invasion within each pT stage. (DOCX 16 kb)

Additional file 2: **Table S2**. Univariate and Multivariate analyses for Lymphovascular Invasion in N0 patients. (DOCX 15 kb)

Additional file 3: **Figure S2**. Recurrence-free survival and Disease-specific survival curves for N0 patients with or without lymphovascular invasion. *LVI: lymphovascular invasion, LVI−/+: negative/positive LVI. (DOCX 34 kb)*

Additional file 4: **Figure S1**. Comparison of the 8th edition of the TNM classification alone and the 8th edition of the TNM classification combined with LVI. *LVI: lymphovascular invasion, LVI−/+: negative/positive LVI. (DOCX 83 kb)*

## Abbreviations

AC-group: Adjuvant chemotherapy group
AIC: Akaike information criteria
BMI: Body mass index
DSS: Disease-specific survival
FMUUH: Fujian Medical University Union Hospital
JCGC: Japanese Classification of Gastric Cancer
LVI: lymphovascular invasion
LVI−/+: Negative/positive LVI
N0: Node-negative
NCCN: National Comprehensive Cancer Network
OS: Overall survival
RFS: Recurrence-free survival
SA-group: Surgery alone group
UICC/AJCC: Union for International Cancer Control/American Joint Committee on Cancer
