# Supplementary material for: Combination of lymphovascular invasion and the AJCC TNM staging system improves prediction of prognosis in N0 stage gastric cancer: results from a high-volume institution
Source: BMC Cancer. 2019 Mar 11;19:216. doi: 10.1186/s12885-019-5416-8 (PMC6413460; doi:10.1186/s12885-019-5416-8)
Supplement: Supplementary file 1 — Table S1. Lymphovascular invasion within each pT stage. (DOCX 16 kb) [file 12885_2019_5416_MOESM1_ESM.docx]

| Supplementary Table 1. Lymphovascular invasion within each pT stage. | | | | |
| --- | --- | --- | --- | --- |
|  | pT1(n=387) | pT2(n=123) | pT3(n=117) | pT4(n=109) |
| LVI－(n) | 355 | 107 | 83 | 85 |
| LVI＋(n) | 32 | 16 | 34 | 24 |
| LVI＋rate(%) | 8.3 | 13.0 | 29.1 | 22.0 |
| *LVI: lymphovascular invasion, LVI-/+: negative/positive LVI* | | | | |
