# Supplementary material for: Combination of lymphovascular invasion and the AJCC TNM staging system improves prediction of prognosis in N0 stage gastric cancer: results from a high-volume institution
Source: BMC Cancer. 2019 Mar 11;19:216. doi: 10.1186/s12885-019-5416-8 (PMC6413460; doi:10.1186/s12885-019-5416-8)
Supplement: Supplementary file 2 — Table S2. Univariate and Multivariate analyses for Lymphovascular Invasion in N0 patients. (DOCX 15 kb) [file 12885_2019_5416_MOESM2_ESM.docx]

| Supplementary Table 2. Univariate and Multivariate analyses for Lymphovascular Invasion in N0 patients | | | | | | |
| --- | --- | --- | --- | --- | --- | --- |
| Variable |  | Univariate |  |  | Multivariate |  |
|  | HR | 95% CI | p Value | HR | 95% CI | p Value |
| Age |  |  | p=0.057 |  |  |  |
| age<55 | Ref | / |  |  |  |  |
| 55≤age<65 | 1.25 | 0.71-2.12 |  |  |  |  |
| age≥65 | 1.8 | 1.09-2.98 |  |  |  |  |
| Sex |  |  | p=0.66 |  |  |  |
| female | Ref | / |  |  |  |  |
| male | 0.89 | 0.54-1.47 |  |  |  |  |
| Tumor location |  |  | p=0.10 |  |  |  |
| upper third | Ref | / |  |  |  |  |
| middle third | 0.79 | 0.44-1.43 |  |  |  |  |
| lower third | 0.61 | 0.38-0.96 |  |  |  |  |
| Tumor differentiation |  |  | p=0.12 |  |  |  |
| well differentiated | Ref | / |  |  |  |  |
| moderately differentiated | 0.72 | 0.45-1.16 |  |  |  |  |
| poorly differentiated or undifferentiated | 0.54 | 0.33-0.98 |  |  |  |  |
| T-stage |  |  | p<0.001 |  |  | p<0.001 |
| T1 | Ref | / |  | Ref | / |  |
| T2 | 1.65 | 0.88-3.13 |  | 1.65 | 0.88-3.13 |  |
| T3 | 4.54 | 2.65-7.78 |  | 4.54 | 2.65-7.78 |  |
| T4 | 3.13 | 1.75-5.59 |  | 3.13 | 1.75-5.59 |  |
