# Supplementary material for: Combination of lymphovascular invasion and the AJCC TNM staging system improves prediction of prognosis in N0 stage gastric cancer: results from a high-volume institution
Source: BMC Cancer. 2019 Mar 11;19:216. doi: 10.1186/s12885-019-5416-8 (PMC6413460; doi:10.1186/s12885-019-5416-8)
Supplement: Supplementary file 3 — Figure S2. Recurrence-free survival and Disease-specific survival curves for N0 patients with or without lymphovascular invasion. LVI: lymphovascular invasion, LVI−/+: negative/positive LVI. (DOCX 34 kb) [file 12885_2019_5416_MOESM3_ESM.docx]

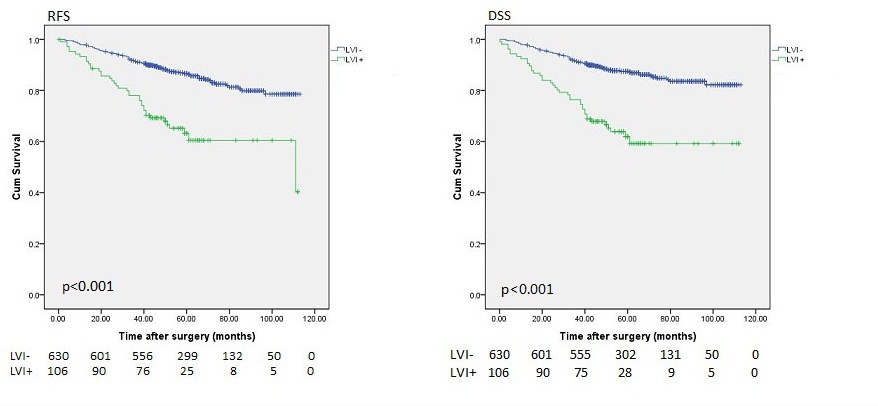


Supplementary Figure 2. Recurrence-free survival and Disease-specific survival curves for N0 patients with or without lymphovascular invasion.*LVI: lymphovascular invasion, LVI-/+: negative/positive LVI.*
