# Supplementary material for: Combination of lymphovascular invasion and the AJCC TNM staging system improves prediction of prognosis in N0 stage gastric cancer: results from a high-volume institution
Source: BMC Cancer. 2019 Mar 11;19:216. doi: 10.1186/s12885-019-5416-8 (PMC6413460; doi:10.1186/s12885-019-5416-8)
Supplement: Supplementary file 4 — Figure S1. Comparison of the 8th edition of the TNM classification alone and the 8th edition of the TNM classification combined with LVI. LVI: lymphovascular invasion, LVI−/+: negative/positive LVI. (DOCX 83 kb) [file 12885_2019_5416_MOESM4_ESM.docx]

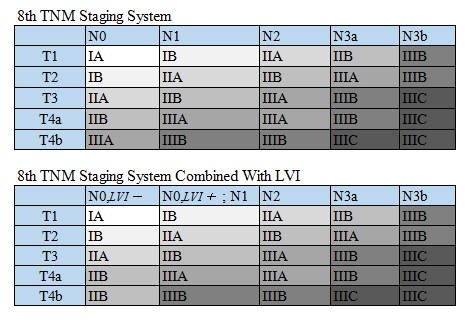


Supplementary Figure 1. Comparison of the 8th edition of the TNM classification alone and the 8th edition of the TNM classification combined with LVI. *LVI: lymphovascular invasion, LVI-/+: negative/positive LVI.*
